# Cranial volume measurement with artificial intelligence and cognitive scales in patients with clinically isolated syndrome

**DOI:** 10.3389/fneur.2024.1500140

**Published:** 2024-12-11

**Authors:** Özlem Albuz, Ibrahim Acir, Ozan Haşimoğlu, Melis Suskun, Elif Hocaoğlu, Vildan Yayla

**Affiliations:** ^1^Bakırköy Dr. Sadi Konuk Eğitim ve Araştırma Hastanesi, Istanbul, Türkiye; ^2^Basaksehir Cam and Sakura City Hospital, Istanbul, Türkiye

**Keywords:** multiple sclerosis, clinically isolated syndrome, artificial intelligence, BrainLab, brain volume analysis

## Abstract

**Objective:**

We aimed to investigate the relationship between volumetric measurements of specific brain regions which were measured with artificial intelligence (AI) and various neuropsychological tests in patients with clinically isolated syndrome.

**Materials and methods:**

A total of 28 patients diagnosed with CIS were included in the study. The patients were administered Öktem Verbal Memory Processes Test, Symbol Digit Modalities Test (SDMT), Backward-Forward Digit Span Test, Stroop Test, Trail Making Test, Controlled Oral Word Association Test (COWAT), Brief Visuospatial Memory Test, Judgement of Line Orientation Test, Beck Depression Scale, Beck Anxiety Scale and Fatigue Severity Scale. Artificial intelligence assisted BrainLab Elements^™^ Atlas-Based Automatic Segmentation program was used for calculating volumes. The measured volumes were compared with the reference database. In addition, neuropsychological test performances and volumetric measurements of the patients were compared.

**Results:**

Of the patients included in the study, 78.6% were female and 21.4% were male, with an average age of 33 years. Verbal Memory Processes Test, SDMT, Backward-Forward Digit Span, JLOT, and Stroop Test showed significant correlations with multiple anatomical regions, particularly the anterior thalamic nucleus, which was associated with the highest number of cognitive tests. The JLOT exhibited the strongest correlation with six different brain regions (*p* < 0.001).

**Conclusion:**

The Judgement of Line Orientation and Stroop Tests, correlated with multiple brain regions, especially the anterior thalamic nucleus, underscoring the importance of these tests in assessing cognitive function in CIS.

## Introduction

Clinically isolated syndrome (CIS) is defined as one of the subtypes of multiple sclerosis (MS) according to the 2017 McDonald MS criteria. It is a monophasic clinical episode suggestive of a focal or multifocal, inflammatory demyelinating event in the central nervous system, lasting at least 24 h, with or without subsequent improvement, not accompanied by infection or fever, and including symptoms resembling a typical MS relapse (1). Although almost any neurological finding may be the first clinical episode in patients with CIS, somatosensory findings, optic neuritis, transverse myelitis, brainstem syndrome, and cognitive involvement are most commonly observed (2, 3). Cognitive impairment was first mentioned by Charcot in 1877 as “slowness in the perception of MS patients.” Cognitive impairment has been reported to be approximately 34–65% (4).

Brain tissue loss (atrophy) is thought to reflect neuroaxonal damage. Volumetric measurements are performed with fully automatic segmentation software over 3D T1-weighted sequences to evaluate atrophy. Atrophy starts in the early period of the disease, and it is known to be strongly associated with cognitive impairment (5).

Cognitive impairments observed in MS include impairments in information processing efficiency and speed, attention maintenance and complex attention, working memory, learning, problem-solving, language and visuospatial memory, long-term memory, abstract thinking, verbal fluency, and executive functions (6). The characteristics of cognitive impairment in the CIS group are similar to those of MS, and information processing speed and verbal memory are most commonly affected. It has been suggested that cognitive dysfunction observed in patients with CIS may predict the transformation of the disease into MS and the disability that occurs over time (7, 8).

The possibility of establishing a correlation between radiological images and cognitive impairment in MS is very important, and many studies have been conducted on this subject. In studies, cognitive impairment was found to be associated with T2 lesion load, neocortical gray matter, volume loss in the thalamus, hippocampus, and corpus callosum on MR imaging (6, 9–11).

Our primary aim encompassed a comprehensive inquiry into the intricate interplay between the volumetric measurements derived from distinct cerebral regions in CIS patients and a diverse array of neuropsychological tests, delving into the nuanced associations and potential implications within this multifaceted relationship.

## Materials and method

In this study, a total of 28 patients comprising 6 males and 22 females diagnosed with CIS, and who were under observation at the demyelinating diseases outpatient clinic between February–June 2023, were assessed. Inclusion criteria stipulated that patients must have been diagnosed with clinically isolated syndrome, be 18 years of age or older, be proficient in Turkish, and exhibit normal laboratory test results concerning cognitive function. Exclusion criteria encompassed substance abuse, recent acute exacerbations or corticosteroid use within 4 weeks before clinical and MR imaging tests, presence of central nervous system diseases, significant affective disorders or severe psychiatric illnesses, utilization of psychostimulant or psychotropic drugs affecting cognitive functions, alcohol or substance dependence, as well as a history of attention deficit-hyperactivity disorder and learning disabilities.

**Figure 1:**
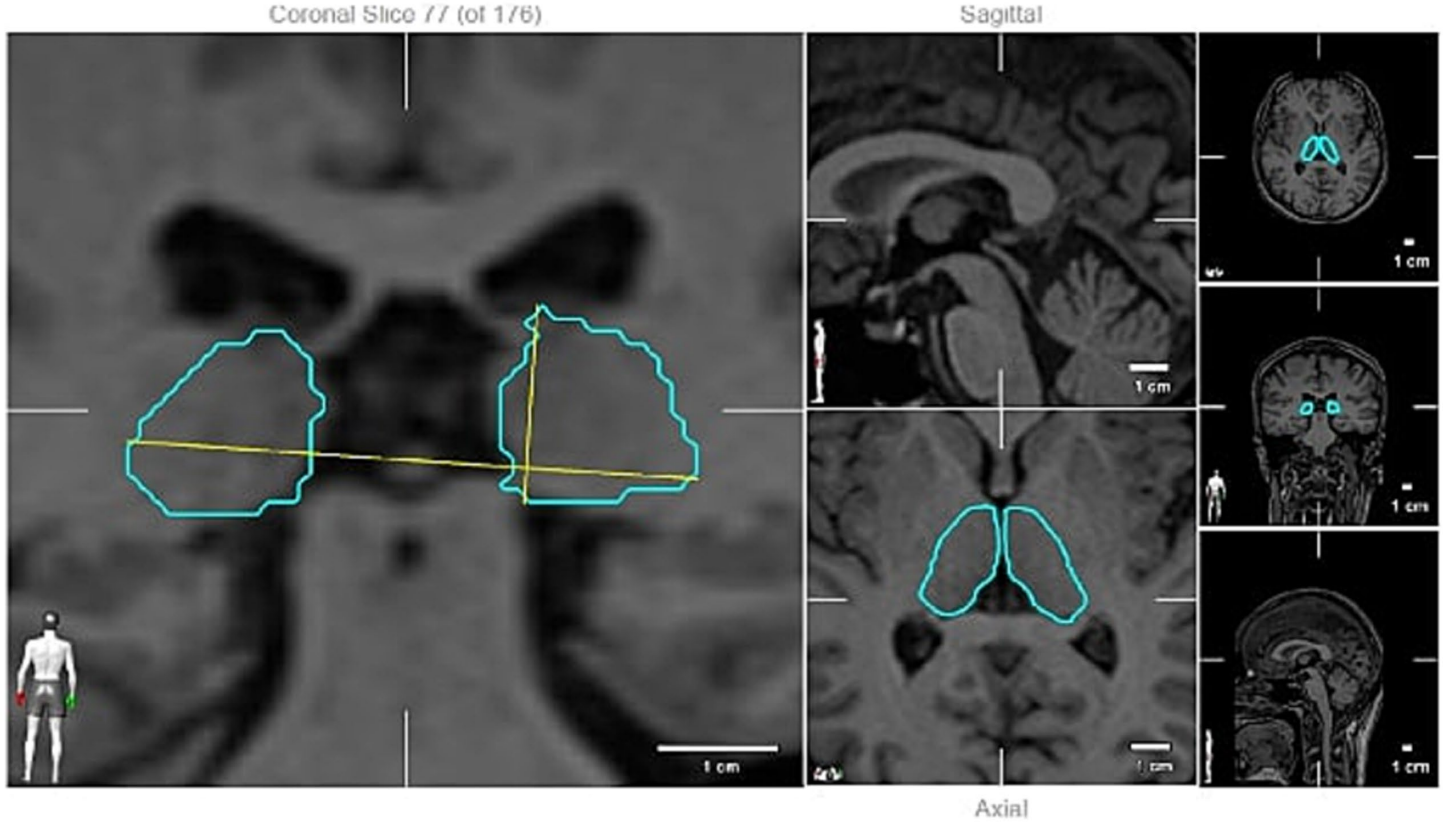
Thalamic volume measuring (an example).

**Figure 2:**
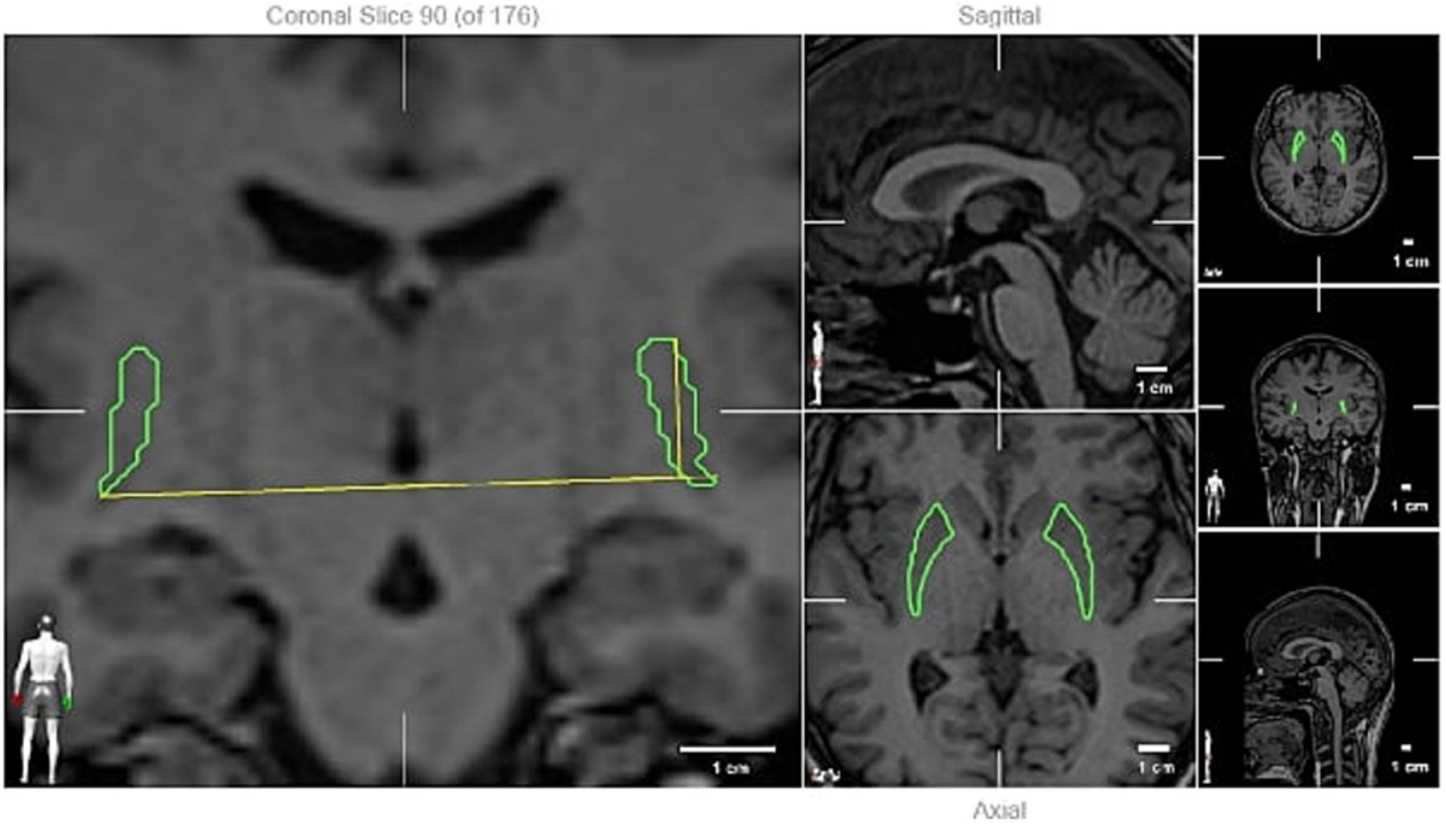
Putamen volume measuring (an example).

Patients underwent cranial MR imaging with a slice interval of 1 mm. The imaging was conducted in the supine position utilizing a 1.5 Tesla magnetic field strength (Siemens Magnetom Amira) device equipped with an 8-channel head coil, adhering to the MS acquisition protocol. All images were acquired using the same device and included Turbo spin echo T1 (TR 1,060 ms, TE Shortest ms, slice thickness 1 mm with no gaps, matrix 252 × 240 pixels) and T2w (TR 2,500 ms, TE: shortest 260 ms, slice thickness 1 mm with no gaps, matrix 252 × 252 pixels) sequences. The radiological images were converted to the appropriate format and transferred to the BrainLab Elements^™^ Atlas-Based Automatic Segmentation program, where the volumes of the patients were evaluated by a certified neurosurgeon trained in volume measurement. In this system, the most accurate boundaries of the grey matter and basal ganglia were automatically identified by comparing the voxel parameters of the patient with the parameters in the atlas averages through artificial intelligence. Subsequently, after the fusion of the T2w and T1 MR images of the patients in the BrainLab Elements program, all grey matter and basal nuclei were automatically segmented separately in the object segmentation module, and their boundaries and volumes were calculated. The boundaries were cross-checked on the T2w image, and any inaccuracies in segmentations were rectified. The volume values obtained were then juxtaposed with the average volume values in the MNI PD25 and ICBM152 standard human brain database (12), and the variance for each anatomical region was recorded. The measured volumes included the amygdala, capsule externa, capsule interna, nucleus caudatus, cerebellum, nucleus dentatus, fornix, globus pallidus, hypothalamus, nucleus accumbens, basal nucleus of Meynert, nucleus ruber, optic nerve, pedunculopontine nucleus, putamen, substantia nigra, anterior thalamic nucleus, zona incerta, and ventricle volumes, which were subsequently compared to the reference database using the BrainLab Elements^™^ Atlas-Based Automatic Segmentation program. The measured volumes of the patients were compared with the reference database (topographic volume-standardization atlas of the human brain) (Figures 1, 2) (13).

Öktem Verbal Memory Processes Test, Paced Auditory Serial Addition Test (PASAT), Symbol Digit Modalities Test (SDMT), Backward-Forward Digit Span Test, Stroop Test, Trail Making Test, Controlled Oral Word Association Test (COWAT), Brief Visuospatial Memory Test (BVMT-R), Judgment of Line Orientation Test (JLOT), Beck Depression Scale, Beck Anxiety Scale, and Fatigue Severity Scale (FSS) neuropsychological tests were administered, which lasted approximately 90 min within 2 weeks following MRI. The PASAT test was only administered to one person due to communication and cooperation difficulties between the patients and the administrator, as well as the challenges in administering the test. Therefore, this test was excluded from the study.

All statistical analyses were performed using IBM SPSS Statistics version 29.0. Descriptive statistics were expressed as mean ± standard deviation (mean ± SD) or median (25th–75th percentile) values for continuous variables and as numbers (*n*) and percentage (%) for categorical variables. The comparison between categorical variables was conducted using the chi-square test or Fisher’s exact test. The determination of normal distribution was based on the number of observations in the groups, histograms, and the Shapiro–Wilk test. The Mann–Whitney *U* test was employed to compare continuous variables that were not normally distributed between two groups. If normal distribution was confirmed, Student’s *t*-test was utilized. The linear relationship between two continuous variables was assessed using Pearson or Spearman correlation coefficients, and their significance was analyzed based on the presence or absence of normal distribution. Correlation coefficients falling between 0 and ± 0.3 were interpreted as indicating no correlation, while coefficients between 0.3 and 0.5 suggested a weak correlation in a positive (or negative) direction. Coefficients ranging from 0.5 to 0.7 indicated a moderate correlation in a positive (or negative) direction, while coefficients exceeding 0.7 were indicative of a strong correlation (positive or negative). In cases where the influence of a third variable was considered, partial correlation coefficients were calculated. Two-way *p*-values less than 0.05 were considered statistically significant.

## Results

Demographic and clinical characteristics of the patients who participated in our study are summarised in Table 1. A total of 22 (78.6%) of the patients were female, while 6 (21.4%) were male. The ages of all patients ranged between 17 and 51 years, with a mean age of 33.0 years. Half of the patients (50%) had less than 8 years of education, while the other half had more than 8 years of education. Clinical attacks manifested as optic neuritis in 15 (53.6%) patients, brainstem symptoms in 4 (14.3%) patients, sensory symptoms in 8 (28.6%) patients, and cerebellar symptoms in 1 (3.57%) patient. Diabetes mellitus (DM) was present in 2 patients, hypertension (HT) was present in 2 patients, and hypothyroidism was present in 1 patient. However, thyroid function tests were within normal limits in all patients according to laboratory tests (Table 1).

**Table 1:** Clinical and demographic characteristics of patients with clinically isolated syndrome included in the study.

	[All] *N* = 28
Gender	
Woman	22 (78.6%)
Male	6 (21.4%)
Age	33.0 [17–51]
Education status	
<8 years	14 (50%)
>8 years	14 (50%)
Marital status	
Married	17 (60.7%)
Single/divorced	11 (39.3%)
Profession	
Not working	16 (57.1%)
Labourer, civil servant, other	12 (42.9%)
BMI	26.5 [19.6; 38.1]
Smoking	10 (35.7%)
Alcohol use	2 (7.14%)
Presence of comorbidities	
DM	2 (7.14%)
HT	3 (10.7%)
Hypothyroidism	1 (3.57%)
Other	3 (10.71%)
First attack pattern	
Optic neuritis	15 (53.6%)
Brain stem	4 (14.3%)
Sensory	8 (28.6%)
Cerebellar	1 (3.57%)

When the volumetric examinations of the patients were compared according to gender, a statistically significant difference was found between the two groups in cerebellum, hypothalamus, nucleus accumbens, periquaductal grey matter and subthalamic nucleus volumes (*p* < 0.05) (Table 2).

**Table 2:** Comparison of volumetric measurements according to gender.

	Female	Male	*p* overall	*N*
	*N* = 22	*N* = 6		
Cerebellum	121 (13.6)	136 (4.32)	0.010	28
Hypothalamus	1.25 (0.14)	1.45 (0.09)	0.003	28
Nucleus accumbens	0.94 (0.12)	1.09 (0.14)	0.022	28
Periacuaductal grey matter	0.24 (0.05)	0.31 (0.04)	0.008	28
Subthalamic nucleus	0.18 (0.02)	0.20 (0.01)	0.039	28

The measured volumes of the patients were compared with the volumes of amygdala, basal ganglia (caudate + putamen + globus pallidus), capsule interna, nucleus caudatus, cerebellum, thalamus, globus pallidus, putamen and ventricle in the reference database. Mean ± SD values and statistical comparisons are shown in Table 3. Amygdala, basal ganglia caudate + putamen + globus pallidus, capsule interna, nucleus caudatus, thalamus, globus pallidus putamen and cerebellum were found to be significantly different from the population mean in the sample group with clinically isolated syndrome (*p* < 0.001).

**Table 3:** Comparison of patient volumes with population averages according to topographic volume-standardisation atlas of the human brain database.

	Mean ± SD (patient)	Mean ± SD (atlas) (ATLAS)	*t* value	*p*
Amigdala	2.87 ± 0.29	3.12 ± 0.47	−4.6187	**<0.001**
Basal ganglia (caudate + putamen + globus pallidus)	19.41 ± 1.20	22.12 ± 2.98	−7.1675	**<0.001**
Capsula interna	9.11 ± 1.04	10.62 ± 1.55	−7.6597	**<0.001**
Caudate nucleus	7.22 ± 10.90	7.78 ± 1.32	−3.3053	**0.003**
Cerebellum	116.73 ± 12.62	124 ± 13.8	−2.901	**0.007**
Ventricle	23.3 ± 5.52	21.18 ± 16.71	1.999	0.06
Thalamus	11.1 ± 1.33	14.61 ± 1.46	−13.89	**<0.001**
Globus pallidus	3.07 ± 0.59	3.69 ± 0.38	−8.5068	**<0.001**
Putamen	8.51 ± 0.93	11.26 ± 1.66	−15.64	**<0.001**

Bold values: highly significant.

When examining the correlation between cognitive tests and anatomical regions, no significant correlation was found with the COWAT, BVMT-R, Beck Depression Scale, Beck Anxiety Scale and FSS tests. However, significant correlations were observed with the Öktem Verbal Memory Processes Test, SDMT, Backward-Forward Digit Span Test, JLOT and Stroop Tests. The JLOT was the test that showed correlations with the most anatomical locations (6 anatomical regions). The anterior thalamic nucleus was identified as the anatomical region that correlated with the highest number of cognitive tests. The statistically significant results of the correlation analyses between the cognitive tests and anatomical region volumetric measurements of the patients are shown in Table 4.

**Table 4:** Correlation analysis between cognitive tests and anatomical region volumetric measurements.

Cognitive test	Anatomic region	Correlations	*p*
Öktem Verbal Memory Processes Test	Subtalamic nucleus	−0.421	0.026
SDMT	Acumbal nucleus	0.376	0.048
SDMT	Anterior thalamic nucleus	0.482	0.009
Trail Making Test	Internal capsule	−0.463	0.013
Trail Making Test	Acumbens nucleus	−0.501	0.007
Trail Making Test	Meynert’s basal nucleus	−0.389	0.040
Trail Making Test	Putamen	−0.435	0.021
Trail Making Test	Talamus	−0.486	0.009
Backward-Forward Digit Span Test	Anterior thalamic nucleus	0.374	0.050
Judgement of Line Orientation Test	Capsule interna	0.515	0.005
Judgement of Line Orientation Test	Dentate nucleus	0.477	0.010
Judgement of Line Orientation Test	Globus pallidus	0.436	0.020
Judgement of Line Orientation Test	Acumbens nucleus	0.541	0.003
Judgement of Line Orientation Test	Anterior talamic nucleus	0.453	0.016
Judgement of Line Orientation Test	Talamus	0.409	0.031
Stroop Test	Capsule interna	−0.413	0.040
Stroop Test	Anterior talamic nucleus	−0.545	0.005
Stroop Test	Nucleus caudatus	−0.400	0.047

The regression analysis revealed distinct patterns in the relationship between age, sex, tracking test performance, and volumetric measurements. For the tracking test, the model demonstrated strong explanatory power, accounting for 54% of the variance in performance. Age emerged as a significant predictor, with increasing age associated with longer tracking times (*B* = 6.361, *p* < 0.001). The standardized coefficient (*β* = 0.738) confirmed age as the most influential factor. In contrast, sex had no statistically significant effect on tracking test performance (*p* = 0.795). The model was statistically significant overall (*F* = 14.678, *p* < 0.001), emphasizing the role of age in predicting tracking performance.

The analysis of brain region volumes, including the amygdala, thalamus, capsula interna, putamen, globus pallidus, and nucleus caudatus, showed limited explanatory power. For the amygdala, the model accounted for only 8.3% of the variance, with neither age (*p* = 0.688) nor sex (*p* = 0.176) significantly influencing its volume. Similarly, the capsula interna volume model explained 11.2% of the variance, with age showing no significant effect (*p* = 0.843) and sex being marginally non-significant (*p* = 0.095), suggesting a potential relationship that may require further investigation.

For the thalamus, the model explained 7.6% of the variance, with neither age (*p* = 0.397) nor sex (*p* = 0.309) demonstrating statistical significance. The putamen model performed poorly, explaining only 1.5% of the variance, with both age (*p* = 0.545) and sex (*p* = 0.980) failing to show significant effects. Similarly, the globus pallidus and nucleus caudatus models explained 6.9 and 6.3% of the variance, respectively, with no significant contributions from age or sex for either region.

## Discussion

Clinical isolated syndrome is a single episode of inflammatory demyelination of the central nervous system suggestive of MS. The main mechanism in the pathophysiology of the disease is thought to involve multifocal inflammation, demyelination, oligodendrocyte loss, reactive gliosis, and axonal degeneration (14). In our study, our primary objective was to assess whether atrophy was present by comparing the measured volumes in specific brain regions of patients with clinically isolated syndrome with those in the reference database (topographic volume-standardization atlas of the human brain). Our secondary objective was to evaluate the correlation between the volumes measured in specific brain regions and the results of various cognition tests assessing different cognitive functions, aiming to determine which cognitive performance is most accurately predicted by volume parameters. Previous research has primarily emphasized the role of subcortical structures like the thalamus and basal ganglia in tasks related to executive functions and memory. However, this study expands the scope by examining a more comprehensive set of cognitive tasks, including visuospatial memory, information processing, and working memory, and their associations with specific brain regions in patients with CIS.

Cognitive impairment, often overlooked in daily practice but with a detrimental impact on the daily life activities of patients, is frequently observed in MS. Studies have shown that the prevalence of cognitive impairment ranges from 40 to 65% and may manifest as early as the initial stages of the disease, including during the CIS period (9, 15). It is understood that demyelinating plaques in the periventricular white matter, axonal loss, and neocortical atrophy play crucial roles in the pathophysiology of cognitive impairment. Zipoli et al. (7) identified cognitive impairment in a significant proportion of patients with CIS and concluded that this had prognostic value in predicting conversion to MS. The pattern of cognitive impairment observed in patients with CIS closely resembles that observed in patients with MS, characterized by reduced information processing speed, impaired working memory, executive functions, and attention deficits (15, 16).

In a study that divided MS patients into 3 clusters according to disability status and compared regional volumes with a healthy control group, the volumes of the thalamus, hypothalamus, putamen, and nucleus caudatus were found to be significantly different. It was thought that the ventral diencephalon underwent early degeneration during the course of MS (17). Similarly, in another study aimed at evaluating the relationship between subcortical grey matter and cognition in RRMS patients, atrophy was most prominent in the nucleus caudatus, globus pallidus, and thalamus (18). Furthermore, a study conducted in patients with CIS revealed atrophy in the thalamus, hypothalamus, putamen, nucleus caudatus, and cerebellum compared to the control group (19). In a longitudinal study with a 1-year follow-up MR imaging of RRMS and CIS patients, it was observed that atrophy developed in the grey matter, including the thalamus, nucleus caudatus, putamen, and brainstem. Deep grey matter volume, especially the thalamus volume, was predictive of cognitive performance and disability progression (20). When we compared the volumes measured in our study with the reference database, we found that the volumes of the amygdala, basal ganglia (nucleus caudatus + putamen + globus pallidus), capsule interna, nucleus caudatus, thalamus, globus pallidus, and putamen were significantly different in our patients. This result aligns with findings from other studies and suggests the development of degeneration and secondary atrophy during the clinically isolated syndrome period. Additionally, one of the unique and robust aspects of our study is the utilization of the artificial intelligence-supported BrainLab measurement method, which enables more precise and accurate measurements compared to the measurement methods commonly used in the literature.

The thalamus plays an important role in cognitive functions including attention, information processing speed and memory (21). Neurodegeneration of thalamic nuclei and connections which develops due to inflammation and cytotoxic damage leads to cognitive impairment. Many studies have concluded that thalamic atrophy develops in the early period of the disease and is a strong indicator of cognitive deficits (20, 22). In a study conducted in RRMS patients, thalamus was found to be associated with visuospatial memory (23). In another study conducted in MS patients, SDMT performance was found to be mostly associated with the thalamus and putamen and it was argued that the thalamus plays an important role in information processing efficiency (24). In a different study, thalamus volume was found to be associated with trail making test, Judgement of Line Orientation Test and SDMT performance and it was concluded that it played an important role in memory, working memory and information processing speed (25). In a study conducted by Houtchens et al. (26) in MS patients, it was suggested that thalamus volume was a significant biomarker for information processing speed and visuospatial memory. In a study conducted in patients with CIS, atrophy of the thalamus, putamen and nucleus caudatus was found and it was concluded that thalamic atrophy was an indicator in cognitive evaluation (19). In our study, a significant atrophy was found in the thalamus volume in patients with CIS compared to the reference database. Our study supports that thalamic atrophy develops even in the early period of MS and even in patients with CIS, as in other studies. The fact that a different method was used in our study instead of the commonly used measurement methods in the literature and the results were found to be similar with other studies indicates that there is a correlation between the results of the measurement methods. In addition, there was a correlation between thalamus volume and the tracking test and Judgement of Line Orientation Test.

It has been shown in many studies that the anterior thalamic nucleus plays an important role in learning and memory (27). In a study evaluating the anterior thalamic nuclei in mice, it was shown that they have roles in different stages of memory (28). In another study, a decrease in episodic memory processes, information processing speed, directed attention, working memory and executive functions performance was observed in correlation with age-related decrease in anterior thalamic volume and secondary atrophy (29). In a 3-year follow-up study in MS patients, the anterior thalamic nucleus was found to be more atrophic in patients with cognitive deterioration than in cognitively preserved patients (30). In a cross-sectional study conducted in MS patients, a relationship was found between cognitive deterioration and focal atrophy of the anterior thalamic nucleus (31). In a study examining all nuclei of the thalamus in detail, SDMT performance was found to be correlated with the volume of the left ventral anterior nucleus (32). In our study, there was a correlation between anterior thalamic nucleus volume and SDMT, Backward-Forward Digit Span Test, Stroop and Judgement of Line Orientation Test performance. The positive correlations observed with the SDMT and Backward-Forward Digit Span Test suggest that this region is actively involved in tasks requiring working memory and information processing speed. In contrast, the negative correlation with the Stroop Test indicates that while the anterior thalamic nucleus is engaged in cognitive control and attention tasks, its activity may decrease as performance on inhibitory control tasks improves. This dual role highlights the complexity of the anterior thalamic nucleus in modulating different aspects of cognition, particularly in tasks that require both rapid information processing and cognitive inhibition. These results provide a nuanced understanding of the anterior thalamic nucleus’ contributions to cognitive functions, especially in patients with cognitive impairments.

The nucleus accumbens is known as the centre of reward and pleasure. It plays a modulatory role in the flow of information between the amygdala, basal ganglia, mesolimbic and dopaminergic regions and the prefrontal cortex. The nucleus accumbens is believed to be associated with the cognitive impairment seen in Alzheimer’s disease. It is thought that dopaminergic system changes frequently observed in Alzheimer’s patients are associated with impaired memory performance and reward processing dysfunctions (33). In a study conducted on mice, it was observed that the nucleus accumbens has an important role in mesocorticolimbic dopamine function and cognition (34). In our study, a statistically significant correlation was found between nucleus accumbens volume and SDMT, trail making and Judgement of Line Orientation Test. Based on this, we can say that nucleus accumbens volume predicts working memory, information processing speed, executive functions and visuospatial memory performance. In our research, we did not find any studies on the relationship between nucleus accumbens volume and cognition tests in patients with CIS. We think that comprehensive studies should be conducted on this subject and these findings are one of the unique aspects of our study.

The capsulae interna coordinates cognitive, motor and sensory pathways. Fibre tracts in the anterior crus are associated with emotion, cognition, decision making and motivation (35). In a study evaluating motor and cognitive disorders with diffusion tensor imaging (DTI) in MS patients, a significant correlation was found between capsular interna DTI metrics and 9-hole peg test and PASAT performance (36). In our study, capsular interna volume was found to be atrophic according to the reference database and a significant correlation was found between capsular interna volume and stroop, trail making and Judgement of Line Orientation Test. This finding suggests that, similar to MS, capsular interna volume plays a role in working memory, information processing speed, executive functions and visuospatial functions. It was thought that cognitive functions were affected in patients with CIS before conversion to MS and that the change in capsular volume could explain this.

The cholinergic neuron population in the basal nucleus of Meynert’s nucleus is involved in learning, long-term memory, control and maintenance of attention. Its degeneration causes various neuropsychiatric disorders. The association between the accumulation of Lewy bodies in the nucleus of Meynert and dementia and the favourable results obtained in dementia with DBS treatment applied to the nucleus of Meynert are proof of this. In the correlation study of BICAMS and volumetric measurement in MS patients, a significant relationship was found between them and predicted cognitive change in follow-up. In addition, the volume of Meynert’s nucleus was found to be associated with lower SDMT score (37). In our study, a significant correlation was found between the performance of the tracking test and the volume of Meynert’s basal nucleus and it was thought to be predictive of working memory, information processing speed and executive functions. In our research, we could not find any study in this direction in patients with CIS. Therefore, we think that comprehensive studies should be conducted on this subject and these findings are one of the unique aspects of our study. In addition, we believe that large-scale double-blind controlled studies are needed to evaluate the effect of early initiation of cholinesterase inhibitor treatment on the protection of patients from cognitive impairment.

Our findings, particularly the significant correlation between the Judgement of Line Orientation Test and six distinct anatomical regions, as well as the association of the anterior thalamic nucleus with working memory and information processing speed, can provide valuable insights for managing CIS patients. These correlations suggest that the anterior thalamic nucleus plays a critical role in multiple cognitive domains, especially those related to visuospatial processing, working memory, and rapid cognitive functioning. For CIS patients, who often experience early neurological symptoms that may precede multiple sclerosis, assessing cognitive functions through specific tests like the Judgement of Line Orientation Test and evaluating the integrity of the anterior thalamic nucleus may offer a more targeted approach for early intervention. For instance, using the Judgement of Line Orientation Test can help assess visuospatial abilities, a domain that may be disrupted in CIS due to early thalamic or parietal lobe involvement. Furthermore, the strong correlation of the anterior thalamic nucleus with working memory and processing speed highlights the importance of monitoring these cognitive functions in CIS patients, as deficits in these areas may signal more extensive brain involvement or the transition to MS. By incorporating these specific tests into routine clinical assessments for CIS patients, healthcare providers can better identify early cognitive changes, tailor cognitive rehabilitation strategies, and potentially intervene earlier in the disease course.

Our study highlights the differential predictive power of age and gender on various brain region volumes and cognitive functions. While age emerged as a significant predictor for tracking test performance, it showed no substantial impact on the volumes of key subcortical structures such as the thalamus, amygdala, and putamen. Similarly, gender demonstrated borderline significance for some regions, such as the capsula interna, but was not a robust predictor overall. These results suggest that volumetric changes in certain brain regions may occur independently of these demographic factors, aligning with the growing understanding that intrinsic disease processes in CIS play a dominant role in neurodegeneration.

There is no comprehensive study of this type in the literature that examines various cognitive functions, cranial volumetric measurements and their correlation in patients with CIS. The strengths of this study are that a homogeneous group was formed, a larger number of anatomical regions that had not been evaluated before were evaluated compared to other studies, more precise and accurate volume measurements were provided by using artificial intelligence with the BrainLab Elements^™^ Atlas-Based Automatic Segmentation programme, and a large number of neuropsychological tests covering the main cognitive functions were used. The limitations of our study are that, it is a single-centre study, cross-sectional evaluation and we did not estimate pre-disease intelligence. The number of CIS patients included in the study is relatively lower compared to MS patients. Additionally, for volumetric analysis to be performed, MR imaging needs to be acquired using consistent techniques and sequences, which further limited the number of eligible patients. This is one of the reasons for the small sample size, which presents a limitation in terms of the generalizability of the results. However, despite this limitation, careful and reliable analyses were conducted using the BrainLab Elements^™^ Atlas-Based Automatic Segmentation program. In addition, the fact that we did not include the anatomical locations of demyelinating lesions in our analyses can be counted as another factor. Future longitudinal studies are needed to determine the usefulness and predictive value of volumetric measurements and cognitive functions in determining the risk of conversion to MS in patients with CIS.

## Conclusion

In conclusion, our study highlights the significant role of the anterior thalamic nucleus in various cognitive functions, particularly in working memory, information processing speed, and visuospatial tasks in patients with CIS. The Judgement of Line Orientation Test emerged as a key tool for assessing visuospatial abilities, demonstrating strong correlations with multiple brain regions in patients with CIS.

## Data Availability

The raw data supporting the conclusions of this article will be made available by the authors, without undue reservation.
